# Health App Possession Among Smartphone or Tablet Owners in Hong Kong: Population-Based Survey

**DOI:** 10.2196/mhealth.7628

**Published:** 2017-06-05

**Authors:** Chen Shen, Man Ping Wang, Joanna TW Chu, Alice Wan, Kasisomayajula Viswanath, Sophia Siu Chee Chan, Tai Hing Lam

**Affiliations:** ^1^ School of Public Health The University of Hong Kong Hong Kong China (Hong Kong); ^2^ School of Nursing The University of Hong Kong Hong Kong China (Hong Kong); ^3^ Center for Community-Based Research, Dana-Farber Cancer Institute/Department of Social and Behavioral Sciences Harvard TH Chan School of Public Health Cambridge, MA United States

**Keywords:** apps, smartphone, Chinese

## Abstract

**Background:**

Health apps are increasingly used with important implications for health. Hong Kong is one of the most technologically advanced and connected cities—smartphone ownership and Internet access rates are among the highest in the world.

**Objective:**

We investigated the prevalence of health app possession and related sociodemographic factors and health behaviors among smartphone or tablet owners in Hong Kong.

**Methods:**

A territory-wide population-based dual (landline and mobile) telephone survey was conducted in 2016. Respondents were asked whether they had health-related apps on their smartphones or tablets and what functions were available on the apps (eg, tracking physical activity and logging health records). Logistic regression was used to calculate the adjusted odds ratio (aOR) and 95% CI of health app possession for different demographic characteristics, socioeconomic position (education, employment, and income), health behaviors (smoking, alcohol, and physical activity) and health (body mass index and chronic diseases).

**Results:**

Of the 4129 smartphone or tablet owners (81.28%, 4129/5080 respondents), 995 (24.10%) had a health app. Tracking physical activity (67.0% of 995) and logging health records (43.0% of 995) were the most common functions of the health apps. Overall, younger age, higher education, and household income were associated with having health apps (all *P*<.001). Compared with physical inactivity, engaging in moderate physical activity ≥1 day/week was associated with having health apps (aOR 1.45 [95% CI 1.20-1.75] for 1-3 days/week, and aOR 1.32 [95% CI 1.07-1.62] for ≥4 days/week). Having a history of chronic diseases was associated with having health apps (aOR 1.36 [95% CI 1.11-1.68]).

**Conclusions:**

We have shown a lower prevalence of use of information and communication technologies (ICTs) in respondents with lower education and income in the most developed Chinese city. This could be seen as a confirmation of the “Inverse information law,” which suggests that those most in need have less use of services and hence receive less benefits from advancements in medicine and health related ICTs.

## Introduction

Globally, the use of smartphones such as Apple’s iPhone and Google’s Android is rapidly increasing. Smartphones enable users to browse websites, check emails, and socialize. Smartphone apps related to health provide new ways to deliver information, strategies, and tracking capabilities related to the self-management of health and well-being [1]. Health apps include a wide range of functions such as lifestyle monitoring, self-diagnosis of disease, and treatment management [2]. The number of health apps is rapidly expanding [3]; the number was 8000 in 2010 and it tripled in 2015 [4].

Interventions using health apps showed effectiveness for weight loss, glycemic control, smoking cessation, and recovery from alcoholism [5-8]. However, reports on the pattern of health app use such as the prevalence of health app possession or the frequency of use in the general population are scarce. One population-based telephone survey in the United States in 2012 reported that about 19% of mobile phone users had a health-related mobile app, while an updated survey in 2015 reported that the above proportion had increased to nearly 60% [9]. People with higher education and household income are more likely to have health apps because they are more health conscious and have more health information orientation and health literacy [2,10]. One potential theoretical framework explaining the socioeconomic inequalities in the possession of health apps is the communication inequality theory, which defines communication inequalities as the differences among social groups in their ability to access, process, and act on information [11]. For instance, education may provide essential knowledge, confidence, and a sense of efficacy in enabling someone to navigate health information communicated by health apps. Discretionary income may allow a person to own a smartphone as well as to access the Internet and purchase data packages and chargeable health apps. Health app possession may also be associated with other demographic factors as well as health behaviors. The possession of health apps among smartphone owners declines with age, likely due to perceived access barriers to information and communication technologies (ICTs) [9,12-14]. Instead, older people are more likely to seek health information using traditional media such as newspapers or radios. Latino/Hispanic people are more likely to have health apps than white people [9]. Furthermore, obesity is also associated with having health apps [9]. Diagnosis of chronic diseases is associated with seeking more health information through the Internet [14].

Social patterning of health app possession may differ by context. Hong Kong is the most modernized and westernized city in China. However, education levels in Hong Kong are much lower than the West as universal education has been introduced only in recent decades [15]. About 16% of Hong Kong’s residents had primary education, and 14% did not receive any formal education [16]. In addition, Hong Kong is a setting with high socioeconomic inequalities and a higher Gini coefficient (0.531 in 2011) than most developed countries [17]. The wide gap between the rich and the poor puts people with a low socioeconomic position (SEP) at a great disadvantage in terms of being able to afford a smartphone or tablet and get access to the Internet. The majority of Hong Kong’s population is of Chinese ethnicity (93.6%), making the impact of race or ethnicity on having health apps less relevant. As such, studies investigating the prevalence and determinants of health app possession in a non-Western setting may help produce contextually specific policies and interventions to promote health apps.

Hong Kong has experienced widespread use of smartphones (about 83.3% of adults have used a smartphone in the past 12 months) and the Internet (about 84.3% of adults have used the Internet in the past 12 months), owing to advanced cyber-infrastructure and the low cost of access to the Internet. The smartphone has replaced the personal computer (78.6%) as the most common Web access device [18]. Smartphone ownership and Internet access rates in Hong Kong are among the highest in the world [19]. To our knowledge, no study has reported on health app possession and related factors in Asia. Hong Kong is one of the most developed non-Western cities, where the sociodemographic characteristics are different from the West but the use of smartphones is similarly prevalent. As the first step, we took advantage of a large population-based telephone survey to investigate the prevalence of health app possession and examine related factors such as sociodemographic factors, health behaviors, body mass index (BMI), and chronic diseases among Hong Kong’s Chinese adults.

## Methods

### Sampling

The Hong Kong Family and Health Information Trends Survey (FHInTS) is part of the FAMILY Project, entitled “FAMILY: a Jockey Club Initiative for a Harmonious Society.” FHInTS is a regular periodic probability-based telephone survey of the general Hong Kong public, designed to assess opinions and behaviors with regard to family health, information use, and health communication. So far, five waves of FHInTS have been conducted since 2009, and details of previous waves were reported elsewhere [14,20]. The current wave was conducted from January to August 2016 to collect data on ICT use for family and health information, family communication, and well-being.

The survey consisted of landline and mobile samples in the proportion of 4:1. All interviews were conducted by trained interviewers from the Public Opinion Program, University of Hong Kong, which is one of the largest established survey agencies, using the Web-based Computer-Assisted Telephone Interview system. The survey targeted the Cantonese-speaking adult population aged 18 years and over. Landline and mobile telephone numbers were randomly generated using known prefixes assigned to telecommunication services providers under the Numbering Plan provided by the Office of the Communications Authority, which covers nearly all Hong Kong residents [21]. For the landline telephone number samples, when contact was successfully established with a target household, a qualified person was selected from all those present using the “next birthday” method [22]. The person from the household who had the next birthday among all household members who were aged 18 years or over was selected as the respondent. No second-level sampling, that is, next birthday rule was used for the mobile sample. Interviews were mostly conducted in the afternoons and evenings (2:00-10:30 PM). Ethical approval was granted by the Institutional Review Board (IRB) of the University of Hong Kong / Hospital Authority Hong Kong West Cluster. Verbal informed consent was obtained from the respondents.

### Measurements

Health app possession was determined by asking the respondents who reported ownership of smartphones or tablets whether they had any software apps related to health. For those who reported having health apps, we asked them whether their apps had the following functions: logging health records (eg, body weight), tracking physical activity (eg, number of steps walked), tracking calorie intake or meals for weight loss, tracking health measures through a wearable device (eg, blood pressure and heart rate), managing specific conditions and diseases, helping quit smoking and alcohol consumption, tracking baby or child health, monitoring sleep, and acquiring health information. We chose these functions because they are common functions of health apps [2,9].

SEP was measured using educational attainment, employment status, and monthly household income. Educational attainment was categorized as primary or below, secondary, and tertiary or above. Employment status was categorized as full-time, part-time, self-employed, and unemployed. Monthly household income was categorized as <HK $10,000, HK $10,000-19,999, HK $20,000-29,999, HK $30,000-39,999, and ≥HK $40,000 (US $1=HK $7.8).

Smoking was categorized as nonsmoker, current smoker, and ex-smoker. Alcohol consumption was categorized as never drinker, occasional drinker (less than once per month), monthly drinker (1-3 days per month), weekly drinker (at least 1 day per week), and ex-drinker. Frequency of moderate physical activity for 10 minutes in the past 7 days was categorized as none, 1-3 days per week, and 4-7 days per week. BMI (weight in kilograms/height in square meters) was classified as <18.5 (underweight), 18.5 to <23 (normal), 23 to <25 (overweight) and ≥25 (obese). History of doctor-diagnosed chronic diseases (cancer, cardiovascular diseases, respiratory diseases, liver diseases, allergies, and others) was classified as none and any. Other information analyzed included sex, age, and marital status.

### Statistical Analysis

To improve the representativeness of the findings, the raw data were weighted using random iterative method [23,24] according to provisional figures obtained from the Census and Statistics Department on the sex-age distribution of the Hong Kong population at the end of 2015 and the educational attainment (highest level attended) distribution in the 2011 census. Chi-square tests were used to assess the differences in sociodemographic characteristics, health behaviors, BMI, and history of diagnosed chronic diseases between smartphone or tablet owners and nonowners. The associations of age, sex, marital status, and SEP with health app possession were analyzed by logistic regression in a model with these variables mutually adjusted and additional adjustment for the mode of survey (landline or mobile). Associations of health behaviors, BMI, and history of diagnosed chronic diseases with health app possession were analyzed in a separate model adjusting for age, sex, marital status, SEP, and the mode of survey. Whether the associations varied by the mode of survey was determined from the heterogeneity across strata and the significance of interaction terms. All analyses were conducted using Stata 13.0 (StataCorp LP, College Station, TX, USA). A *P* value of less than .05 was considered statistically significant.

## Results

Of 6890 eligible adults, 5080 were successfully interviewed with a response rate of 73.73% (71.32% (1042/1461) for the mobile survey and 74.38% (4038/5429) for the landline survey). Table 1 shows that of the 5080 respondents, after weighting, 54.89% (2789/5080) were women, 72.32% (3673/5080) were aged 25 to 64 years, and 63.12% (3206/5080) were married or cohabitating. Most respondents (76.34%, 3878/5080) had secondary or higher education and 42.39% (1911/4508) had a monthly household income of HK $30,000 or more (the median monthly household income in Hong Kong was HK $25,000 in 2016) [25]. Only a small proportion of the respondents were current smokers (11.24%, 571/5078) or weekly drinkers (9.76%, 496/5079). More than half (55.02%, 2792/5075) were physically inactive and 41.93% (1654/3945) were overweight or obese (BMI>23). Less than one-third (31.69%, 1610/5080) had a history of diagnosed chronic diseases. Additionally, 19.58% of the respondents had a health app (995/5080).

Table 2 shows that smartphone or tablet owners were younger and had higher educational attainment and household income than nonowners. Smartphone or tablet owners were also more physically active and had less chronic diseases than nonowners. 24.10% (995/4129) of smartphone or tablet owners had health apps.

**Table 1:** Sociodemographic characteristics, health behaviors, body mass index, diagnosed chronic diseases, and health app possession of the respondents (n=5080).

Demographics		Unweighted, n (%)	Weighted, n (%)
**Sex**			
	Male	2080 (40.94)	2291 (45.11)
	Female	3000 (59.06)	2789 (54.89)
**Age**			
	18-24	706 (13.90)	481 (9.47)
	25-34	561 (11.04)	879 (17.32)
	35-44	594 (11.70)	921 (18.13)
	45-54	841 (16.56)	983 (19.35)
	55-64	985 (19.39)	890 (17.52)
	65 or above	1393 (27.42)	926 (18.23)
**Marital status**			
	Single	1386 (27.28)	1358 (26.73)
	Married/cohabitating	3069 (60.41)	3206 (63.12)
	Divorced/widowed	625 (12.30)	516 (10.15)
**Educational attainment**			
	Primary or below	1008 (19.84)	1202 (23.66)
	Secondary	2131 (41.95)	2443 (48.09)
	Tertiary or above	1941 (38.21)	1435 (28.25)
**Employment status**			
	Full-time	1610 (31.69)	1948 (38.34)
	Part-time	411 (8.09)	441 (8.68)
	Self-employed	225 (4.43)	274 (5.40)
	Unemployed	2834 (55.79)	2417 (47.58)
**Monthly household income (HK$)**			
	<10,000	1037 (23.00)	889 (19.72)
	10,000-19,999	775 (17.19)	862 (19.12)
	20,000-29,999	767 (17.01)	846 (18.77)
	30,000-39,999	604 (13.40)	624 (13.84)
	40,000 or above	1325 (29.39)	1287 (28.55)
**Smoking status**			
	Nonsmoker	4155 (81.82)	3974 (78.25)
	Current smoker	432 (8.51)	571 (11.24)
	Ex-smoker	491 (9.67)	533 (10.50)
**Alcohol use**			
	Never	2481 (48.85)	2413 (47.51)
	Occasional	1361 (26.80)	1360 (26.77)
	1-3 days/month	563 (11.08)	586 (11.53)
	1 day or more/week	453 (8.92)	496 (9.76)
	Ex-drinker	221 (4.35)	225 (4.43)
			
**Moderate physical activity**			
	None	2763 (54.44)	2792 (55.02)
	1-3 days/week	1158 (22.82)	1151 (22.69)
	4 days or more/week	1154 (22.74)	1132 (22.29)
**Body mass index**			
	<18.5	422 (10.70)	380 (9.63)
	18.5-<23	1951 (49.46)	1911 (48.44)
	23-<25	738 (18.71)	738 (18.72)
	>25	834 (21.14)	916 (23.21)
**Diagnosed chronic diseases**			
	Yes	1813 (35.69)	1610 (31.69)
	No	3267 (64.31)	3470 (68.31)
>**Health app possession**			
	Yes	975 (19.19)	995 (19.58)
	No	4105 (80.81)	4085 (80.42)

**Table 2:** Sociodemographic characteristics, health behaviors, body mass index, diagnosed chronic diseases, and health app possession of respondents who owned smartphones or tablets.

Demographics		Smartphone or tablet owners (n=4129), n (%)	Smartphone or tablet nonowners (n=951), n (%)	*P* value^a^
**Sex**				
	Male	1733 (41.97)	347 (36.5)	
	Female	2396 (58.03)	604 (63.5)	.002
**Age**				
	18-24	704 (17.05)	2 (0.2)	
	25-34	559 (13.54)	2 (0.2)	
	35-44	582 (14.10)	12 (1.3)	
	45-54	792 (19.18)	49 (5.2)	
	55-64	825 (19.98)	160 (16.8)	
	65 or above	667 (16.15)	726 (76.3)	<.001
**Marital status**				
	Single	1324 (32.07)	62 (6.5)	
	Married/cohabitating	2483 (60.14)	586 (61.6)	
	Divorced/widowed	322 (7.80)	303 (31.9)	<.001
**Educational attainment**				
	Primary or below	449 (10.87)	559 (58.8)	
	Secondary	1823 (44.15)	308 (32.4)	
	Tertiary or above	1857 (44.97)	84 (8.8)	
**Employment status**				
	Full-time	1554 (37.64)	56 (5.9)	<.001
	Part-time	368 (8.91)	43 (4.5)	
	Self-employed	219 (5.30)	6 (0.6)	
	Unemployed	1988 (48.15)	846 (89.0)	
**Monthly household income (HK$)**				
	<10,000	529 (14.30)	508 (62.8)	
	10,000-19,999	648 (17.52)	127 (15.7)	
	20,000-29,999	673 (18.19)	94 (11.6)	
	30,000-39,999	570 (15.41)	34 (4.2)	
	40,000 or above	1279 (34.58)	46 (5.7)	<.001
**Smoking status**				
	Nonsmoker	3416 (82.77)	739 (77.7)	
	Current smoker	357 (8.65)	75 (7.9)	
	Ex-smoker	354 (8.58)	137 (14.4)	<.001
**Alcohol use**				
	Never	1850 (44.82)	631 (66.4)	
	Occasional	1224 (29.65)	137 (14.4)	
	1-3 days/month	532 (12.89)	31 (3.3)	
	1 day or more/week	403 (9.76)	50 (5.3)	
	Ex-drinker	119 (2.88)	102 (10.7)	<.001
**Moderate physical activity**				
	None	2103 (50.99)	660 (69.4)	
	1-3 days/week	1077 (26.12)	81 (8.5)	
	4 days or more/week	944 (22.89)	210 (22.1)	<.001
**Body mass index**				
	<18.5	321 (10.30)	101 (12.2)	
	18.5-<23	1575 (50.55)	376 (45.4)	
	23-<25	576 (18.49)	162 (19.5)	
	>25	644 (20.67)	190 (22.9)	.06
**Diagnosed chronic diseases**				
	Yes	1223 (29.62)	590 (62.0)	
	No	2906 (70.38)	361 (38.0)	<.001
**Health app possession**				
	Yes	995 (24.10)	0 (0.0)	
	No	3134 (75.90)	951 (100.0)	<.001

^a^*P* for two-sided chi-square tests.

**Table 3:** Prevalence (weighted) of health app possession (n=995).

Functions of health apps	Prevalence, n (%)
Track physical activity (eg, number of steps walked)	667 (67.0)
Log health records (eg, body weight)	428 (43.0)
Track health measures (eg, heart rate and blood pressure)	300 (30.2)
Manage specific conditions and diseases	206 (20.7)
Track calories or meals for weight loss	178 (17.9)
Others^a^	92 (9.2)

^a^Others included tracking baby or child health (3.5%, 35/995), acquiring health information (2.9%, 29/995), monitoring sleep (2.4%, 24/995), helping quit smoking (0.7%, 7/995) and alcohol consumption (0.5%, 5/995).

Table 3 shows that common functions of health apps included tracking physical activity (67.0%, 667/995), logging health records (43.0%, 428/995), tracking health measures (30.2%, 300/995), managing diseases (20.7%, 206/995) and tracking calorie intake (17.9%, 178/995). Other functions included tracking baby or child health (3.5%, 35/995), acquiring health information (2.9%, 29/995), monitoring sleep (2.4%, 24/995), helping quit smoking (0.7%, 7/995) and alcohol consumption (0.5%, 5/995).

Table 4 shows that health app possession was generally similar between men and women, except that fewer women had apps for tracking physical activity than men (aOR=0.75 [95% CI 0.62-0.91]). Health app possession decreased with age (*P* for trend <.001). Higher education level and household income were associated with having health apps (both *P* for trend <.001). Patterns of associations of age and education with having health apps were similar across health apps for different functions. Higher household income was also associated with having health apps for tracking physical activity (*P* for trend=.004).

Table 5 shows that compared with physical inactivity, engaging in moderate physical activity more than once per week was associated with having health apps (aOR=1.45 [95% CI 1.20-1.75] for 1-3 days/week, and aOR=1.32 [95% CI 1.07-1.62] for ≥ 4 days/week). The patterns were similar across health apps with different functions. Having a history of diagnosed chronic diseases was associated with having health apps (aOR=1.36 [95% CI 1.11-1.68]), having apps for tracking physical activity (aOR=1.48 [95% CI 1.16-1.89]), and having apps for tracking calorie intake (aOR=1.55 [95% CI 1.02-2.38]). The associations of smoking, alcohol use, and BMI with health app possession were less marked. None of the associations varied by mode of survey (all *P* values for interactions >.05).

**Table 4:** Adjusted association of sociodemographic characteristics with health app possession among smartphone or tablet owners (n=4129; adjusted for mode of survey and all variables in this table were mutually adjusted).

Demographic characteristics		Overall (n=995), aOR (95% CI)	*P* value	Track physical activity (n=667), aOR (95% CI)	*P* value	Log health records (n=428), aOR (95% CI)	*P* value	Track health measures (n=300), aOR (95% CI)	*P* value	Manage diseases (n=206), aOR (95% CI)	*P* value	Track calories (n=178), aOR (95% CI)	*P* value
**Sex**													
	Men	1		1		1		1		1		1	
	Women	0.90 (0.76-1.07)	.23	0.75 (0.62-0.91)	.004	1.06 (0.83-1.35)	.65	0.91 (0.70-1.19)	.50	0.91 (0.65-1.26)	.56	1.17 (0.82-1.66)	.38
**Age**			<.001		<.001		<.001		.007		.02		.001
	18-24	1		1		1		1		1		1	
	25-34	1.17 (0.87-1.56)	.30	0.95 (0.69-1.31)	.76	1.12 (0.75-1.66)	.59	1.04 (0.66-1.62)	.88	1.61 (0.92-2.82)	.10	1.11 (0.63-1.95)	.71
	35-44	0.99 (0.71-1.39)	.96	0.96 (0.66-1.40)	.84	1.11 (0.69-1.78)	.66	1.24 (0.74-2.08)	.42	1.32 (0.68-2.57)	.41	0.98 (0.50-1.91)	.95
	45-54	0.75 (0.53-1.05)	.10	0.68 (0.46-0.99)	.04	0.45 (0.27-0.76)	.002	1.03 (0.60-1.75)	.92	1.09 (0.56-2.14)	.79	0.58 (0.28-1.19)	.14
	55-64	0.42 (0.29-0.61)	<.001	0.36 (0.23-0.56)	<.001	0.30 (0.17-0.53)^c^	<.001	0.44 (0.23-0.84)	.01	0.48 (0.22-1.04)	.06	0.35 (0.15-0.78)	.01
	65 or above	0.44 (0.29-0.67)	<.001	0.29 (0.17-0.50)	<.001	0.28 (0.14-0.53)	<.001	0.52 (0.26-1.07)	.08	0.65 (0.28-1.47)	.30	0.36 (0.14-0.93)	.03
**Education attainment**			<.001		<.001		<.001		<.001		.06		.006
	Primary or below	1		1		1		1		1		1	
	Secondary	1.70 (1.10-2.63)	.02	2.17 (1.11-4.23)	0.02	1.13 (0.56-2.27)	.73	2.75 (0.98-7.76)	.06	2.02 (0.78-5.25)	.15	7.15 (0.96-53.1)	.06
	Tertiary or above	2.88 (1.83-4.52)	<.001	3.82 (1.94-7.53)	<.001	2.58 (1.27-5.24)	.009	5.62 (1.97-16.0)	.001	2.56 (0.96-6.85)	.06	10.3 (1.37-77.9)	.02
**Employment status**													
	Full-time	1		1		1		1		1		1	
	Part-time	1.00 (0.74-1.36)	.99	0.90 (0.63-1.29)	.57	0.82 (0.52-1.29)	.39	0.85 (0.51-1.41)	.52	1.01 (0.54-1.87)	.98	0.66 (0.33-1.34)	.25
	Self-employed	0.94 (0.66-1.33)	.71	1.01 (0.68-1.51)	.96	0.94 (0.55-1.60)	.83	1.08 (0.64-1.83)	.78	1.01 (0.51-2.00)	.99	1.09 (0.53-2.23)	.82
	Unemployed	0.94 (0.76-1.16)	.56	0.89 (0.69-1.15)	.36	0.87 (0.64-1.19)	.39	0.85 (0.60-1.20)	.35	1.03 (0.67-1.60)	.88	0.95 (0.61-1.48)	.81
**Monthly household income (HK$)**			<.001		.004		.21		.19		.14		.40
	<10,000	1		1		1		1		1		1	
	10,000-19,999	0.94 (0.65-1.36)	.76	1.04 (0.66-1.66)	.85	1.15 (0.65-2.03)	.63	0.71 (0.37-1.37)	.31	0.37 (0.18-0.78)	0.008	1.00 (0.43-2.34)	.99
	20,000-29,999	1.25 (0.87-1.78)	.22	1.34 (0.86-2.10)	.20	1.29 (0.74-2.25)	.36	1.32 (0.73-2.40)	.36	0.61 (0.32-1.17)	.13	1.54 (0.69-3.42)	.29
	30,000-39,999	1.28 (0.89-1.85)	.19	1.38 (0.87-2.18)	.17	1.27 (0.72-2.23)	.41	1.19 (0.65-2.20)	.57	0.73 (0.38-1.40)	.34	1.18 (0.51-2.71)	.70
	40,000 or above	1.53 (1.09-2.16)	.01	1.58 (1.03-2.44)	.04	1.38 (0.81-2.36)	.24	1.17 (0.65-2.10)	.59	0.87 (0.48-1.59)	.65	1.36 (0.62-2.98)	.44
**Marital status**													
	Single	1		1		1		1		1		1	
	Married/cohabitated	0.95 (0.74-1.21)	.66	0.79 (0.60-1.05)	.10	1.08 (0.76-1.54)	.66	0.87 (0.59-1.27)	.47	0.87 (0.54-1.38)	.55	0.98 (0.60-1.61)	.94
	Divorced/widowed	0.58 (0.36-0.95)	.03	0.58 (0.31-1.09)	.09	0.68 (0.30-1.52)	.35	0.79 (0.36-1.73)	.56	0.37 (0.12-1.13)	.08	0.70 (0.23-2.14)	.53

**Table 5:** Adjusted associations of health behaviors, body mass index, and diagnosed chronic diseases with health app possession among smartphone or tablet owners (n=4129; adjusted for age, sex, marital status, education, employment, income, mode of survey; all variables in this table were mutually adjusted).

Demographic characteristics		Overall (n=995), aOR (95% CI)	*P* value	Track physical activity (n=667), aOR (95% CI)	*P* value	Log health records (n=428), aOR (95% CI)	*P* value	Track health measures (n=300), aOR (95% CI)	*P* value	Manage diseases (n=206), aOR (95% CI)	*P* value	Track calories (n=178), aOR (95% CI)	*P* value
**Smoking status**													
	Nonsmoker	1		1		1		1		1		1	
	Current smoker	0.89 (0.65-1.21)	.46	0.88 (0.62-1.26)	.48	0.81 (0.49-1.33)	.40	0.77 (0.46-1.28)	.31	0.87 (0.46-1.66)	.68	0.82 (0.42-1.62)	.57
	Ex-smoker	1.05 (0.77-1.42)	.77	1.29 (0.91-1.83)	.15	1.07 (0.67-1.71)	.78	1.08 (0.67-1.75)	.75	0.90 (0.48-1.70)	.75	1.06 (0.54-2.06)	.87
**Alcohol use**													
	Never	1		1		1		1		1		1	
	Occasional	1.09 (0.90-1.32)	.40	1.19 (0.94-1.49)	.14	1.17 (0.88-1.54)	.28	1.23 (0.90-1.69)	.19	1.40 (0.97-2.02)	.07	0.72 (0.47-1.10)	.13
	1-3 days/month	1.13 (0.88-1.44)	.34	1.25 (0.95-1.66)	.12	1.18 (0.83-1.67)	.35	1.32 (0.90-1.93)	.16	0.93 (0.56-1.54)	.77	1.24 (0.78-1.98)	.37
	1 day or more/week	0.94 (0.70-1.26)	.66	1.35 (0.98-1.88)	.07	1.12 (0.73-1.72)	.62	1.48 (0.95-2.30)	.08	0.85 (0.45-1.57)	.60	1.33 (0.77-2.33)	.31
	Ex-drinker	0.95 (0.55-1.65)	.87	0.99 (0.50-1.94)	.97	0.72 (0.28-1.87)	.50	0.97 (0.37-2.52)	.95	0.26 (0.04-1.95)	.19	0.26 (0.03-1.90)	.18
**Moderate physical activity**													
	None	1		1		1		1		1		1	
	1-3 days/week	1.45 (1.20-1.75)	<.001	1.46 (1.18-1.82)	.001	1.53 (1.17-2.01)	.002	1.50 (1.10-2.03)	.009	1.74 (1.21-2.50)	.003	1.84 (1.25-2.70)	.002
	4-7 days/week	1.32 (1.07-1.62)	.008	1.21 (0.95-1.55)	.13	1.45 (1.07-1.96)	.02	1.72 (1.24-2.37)	.001	1.32 (0.87-2.01)	.20	1.49 (0.96-2.32)	.08
**Body mass index**													
	18.5-<23	1		1		1		1		1		1	
	<18.5	1.18 (0.86-1.61)	.31	1.05 (0.73-1.51)	.79	1.22 (0.82-1.81)	.32	0.89 (0.53-1.48)	.65	1.42 (0.82-2.44)	.21	1.06 (0.58-1.95)	.84
	23-<25	0.91 (0.69-1.19)	.49	0.93 (0.67-1.28)	.65	1.19 (0.83-1.70)	.34	0.95 (0.61-1.46)	.80	0.74 (0.42-1.28)	.28	1.42 (0.86-2.36)	.17
	>25	1.09 (0.84-1.42)	.52	1.09 (0.80-1.49)	.60	1.19 (0.82-1.71)	.36	1.08 (0.70-1.65)	.73	1.10 (0.67-1.79)	.72	0.81 (0.44-1.50)	.51
**Diagnosed chronic diseases**													
	No	1		1		1		1		1		1	
	Yes	1.36 (1.11-1.68)	.003	1.48 (1.16-1.89)	.001	1.28 (0.94-1.74)	.12	1.24 (0.89-1.73)	.20	1.21 (0.81-1.82)	.35	1.55 (1.02-2.38)	.04

## Discussion

### Principal Findings

To our knowledge, this study has provided the first evidence of health app possession in one of the most developed non-Western urban settings with highly prevalent ownership of smartphones or tablets. Less than one-quarter (24.10%) of smartphone or tablet owners had a health-related mobile app on their devices. The proportion of health app possession in the total sample was 19.58%. Tracking physical activity, health records, and health measures were common functions of health apps. Respondents who were younger and had higher education and household income were more likely to have health apps. Health app possession was less patterned by lifestyle factors, with only physical activity clearly associated with health app possession. The associations were roughly consistent for health apps with different functions.

Our findings are consistent with previous national surveys in the United States, which showed that people who were younger and had higher educational levels and income were more likely to have health apps [2,9]. Our study adds to existing research by reporting that the associations of these factors with apps for different functions were similar. Older people are less likely to have health apps perhaps because of perceived and practical barriers to new technologies [26,27]. Instead, they tend to seek health information from traditional mass media [14]. However, traditional mass media cannot track real-time health conditions or monitor health behaviors. People with high SEP may be more health conscious and have higher health literacy, while also making more use of health apps [10,28]. However, respondents with low SEP are less likely to have health apps because effective and attractive health apps in the marketplace often cost money to download or use, or need wearable devices such as smart wristbands [29], which may be unaffordable to them. Such people often have poor health status and have greater needs to improve their health [30,31]. Our study also adds to existing research by showing that health app possession was less likely among physically inactive respondents, perhaps also because of a lack of health consciousness or motivation [32]. However, these people also have greater needs to resume regular physical activity, and mobile apps have been reported as an effective way to achieve this [5,33]. Thus we have shown the possible emergence of an ICT use pattern that could be a modern example of the “Inverse Care Law” [34] and “Inverse Information Law” [35,36] in ICTs, which suggests that those most in need in the community may have less care and use of services and hence receive less benefits from advancements in medicine and health related ICTs.

The magnitude of the association of education with health app possession is bigger than in the United States [9], possibly explained by the lower education levels and greater socioeconomic inequalities in Hong Kong. People with extremely low education levels may have difficulties in reading or understanding health information communicated by advanced technologies, suggesting a great potential to improve health communication through health apps in disadvantaged groups when health apps are made easier to use, confirmed to have health benefits, and effectively promoted with greater accessibility at lower costs.

Notably, the possession of health apps for tracking calories or meals for weight loss is less prevalent than in the West [9]. However, we found that while the percentage of overweight and obese respondents in this sample (BMI>23) was more than 40%, the association of BMI with health app possession was not evident. Self-monitoring of dietary intake, which is a systematic observation and recording to increase individuals’ awareness of eating behaviors and food consumed, is one of the key components of behavioral weight loss strategies [37]. Several randomized controlled trials in the United States have found that apps for monitoring calorie intake had a good acceptability and feasibility as well as effectiveness in weight loss, because of low time-consumption, expenditure, and intensity [5,38,39].

In addition, the associations of smoking and alcohol use with health app possession were less marked, possibly because of the lack of apps for smoking cessation or alcohol quitting. Less than 1% of the respondents reported having health apps with the function of smoking cessation, although the prevalence of current smokers was 10.4% in Hong Kong in 2015 [18]. Randomized or quasi-randomized controlled trials reported that quit-smoking text-message programs can increase the rate of quitting [40,41]. However, current apps for smoking cessation have low levels of adherence to evidence-based guidelines such as lack of practical advice on how to quit/how not to relapse and the absence of text message alerts [42].

### Limitations

Our study has some limitations. First, we only had information on whether the respondents had health apps on their smartphones or tablets. Given that a few health apps are already installed when a smartphone is purchased, the possession of health apps could not fully represent download or actual use. Second, the information on health app possession was obtained based on self-report, which could be subject to recall bias. Third, given the nature of the cross-sectional design, we could not determine the temporal sequence of health behaviors and the possession of health apps. Health apps may alter inactive lifestyle, which may lead to reverse causality.

### Future Work

Our study suggests several avenues for future research. More detailed information on health app use such as the frequency of use, the reasons for not using the apps, the notifications or the reliability of information provided by health apps should be collected for a better understanding of their low popularity among Hong Kong’s Chinese adults. Studies to further examine the characteristics of health app nonpossessors and nonusers, how they fit in with the “Inverse Information Law,” and whether or not the “Inverse ICT Law” is emerging are needed in other countries and regions. Further studies are also warranted to identify ways to motivate people to download and use health apps actively to track health, especially for those who are in need of the functions that these apps offer. In addition, as many health apps have been launched for commercial purposes, a more comprehensive evaluation is needed to determine whether such apps are evidence-based, effective, user-friendly, and can provide accurate information [43]. Finally, further studies to develop effective methods for the promotion and use of apps for weight loss or smoking cessation are also warranted.

### Conclusions

To our knowledge, this is the first study to provide evidence on the pattern of health app possession in an under-studied developed non-Western setting with high rates of smartphone ownership and Internet coverage. The prevalence of health app possession among smartphone or tablet owners was low in this population, raising the necessity for obtaining a deeper insight into the plausible reasons. Moreover, socioeconomic inequalities and behavioral clustering of health app possession suggested that more resources are needed to promote download and use of health apps after comprehensive evaluation, particularly in disadvantaged groups who are in need of the functions offered by these apps. We have shown a lower prevalence of the use of ICTs among those with lower education and income in the most developed Chinese city. This could indicate the emergence of an “Inverse ICT Law,” which suggests that those most in need may have less use of services and hence receive fewer health benefits communicated by ICTs.

## Abbreviations

aOR: adjusted odds ratio
BMI: body mass index
FHInTS: Family and Health Information Trends Survey
ICT: information and communication technologies
IRB: Institutional Review Board
SEP: socioeconomic position
